# Densely PEGylated Polybenzofulvene Brushes for Potential Applications in Drug Encapsulation

**DOI:** 10.3390/pharmaceutics10040234

**Published:** 2018-11-15

**Authors:** Marco Paolino, Giorgio Grisci, Federica Castriconi, Annalisa Reale, Germano Giuliani, Alessandro Donati, Claudia Bonechi, Gianluca Giorgi, Raniero Mendichi, Daniele Piovani, Antonella Caterina Boccia, Maurizio Canetti, Filippo Samperi, Sandro Dattilo, Cinzia Scialabba, Mariano Licciardi, Eugenio Paccagnini, Mariangela Gentile, Andrea Cappelli

**Affiliations:** 1 Dipartimento di Biotecnologie, Chimica e Farmacia (Dipartimento di Eccellenza 2018–2022), Università degli Studi di Siena, Via Aldo Moro 2, 53100 Siena, Italy; paomar@oneonline.it (M.P.); giorgio.grisci@outlook.it (G.G.); chicca268@hotmail.it (F.C.); reale5@student.unisi.it (A.R.); germix67@hotmail.com (G.G.); alessandro.donati@unisi.it (A.D.); claudia.bonechi@unisi.it (C.B.); gianluca.giorgi@unisi.it (G.G.); 2 Istituto per lo Studio delle Macromolecole (CNR), Via A. Corti 12, 20133 Milano, Italy; raniero.mendichi@ismac.cnr.it (R.M.); piovani@ismac.cnr.it (D.P.); antonella.boccia@ismac.cnr.it (A.C.B.); canetti@ismac.cnr.it (M.C.); 3 Istituto per i Polimeri, Compositi e Biomateriali (IPCB) U.O.S. di Catania, CNR, Via Gaifami 18, 95126 Catania, Italy; fsamperi@unict.it (F.S.); sandro.dattilo@cnr.it (S.D.); 4 Dipartimento di Scienze e Tecnologie Biologiche, Chimiche e Farmaceutiche (STEBICEF), Università degli Studi di Palermo, Via Archirafi 32, 90123 Palermo, Italy; cinzia.scialabba@unipa.it (C.S.); mariano.licciardi@unipa.it (M.L.); 5 Dipartimento di Scienze della Vita, Università degli Studi di Siena, Via Aldo Moro 2, 53100 Siena, Italy; paccagnini@unisi.it (E.P.); mariangela.gentile21@gmail.com (M.G.)

**Keywords:** PEGylation, grafting onto, polybenzofulvene, nanocarrier, drug delivery systems, spontaneous polymerization, affinity polymerization

## Abstract

The technique of grafting side chains onto a linear polymeric backbone is commonly used to confer to the new polymeric material with desired properties, such as tunable solubility, ionic charge, biocompatibility, or specific interactions with biological systems. In this paper, two new polybenzofulvene backbones were assembled by spontaneous polymerization of the appropriate benzofulvene monomers (4,6-PO-**BF3k** and 4’,6-PO-**BF3k**) bearing two clickable propargyloxy groups in different positions of the 3-phenylindene scaffold. Poly-4,6-PO-**BF3k** and poly-4’,6-PO-**BF3k** were grafted with monomethyl oligo(ethylene glycol) (MOEG) to prepare two new polybenzofulvene brushes (i.e., poly-4,6-MOEG-9-TM-**BF3k** and poly-4’,6-MOEG-9-TM-**BF3k**) by means of a “grafting onto” approach, that were characterized from the point of view of their macromolecular features, aggregation liability, and in a preliminary evaluation of biocompatibility. The obtained results make these PEGylated polybenzofulvene brushes (PPBFB) derivatives potentially useful as nanocarriers for nanoencapsulation and delivery of drug molecules.

## 1. Introduction

The dense grafting of polymeric side chains onto a linear polymeric backbone produces a unique class of macromolecules called molecular brushes, polymer brushes, or cylindrical brushes [1,2]. Structural features, such as side chain length, grafting density, and the physicochemical properties of the components (backbone and side chains) confer peculiar features to these macromolecules. Moreover, they may be sensitive to the surrounding environment since their conformational behavior can be affected by solvent properties, temperature, pH, and ionic strength [3]. The synthesis of brush-like architectures can be performed by means of three different approaches [3]: (a) grafting through (GT, polymerization of macromonomers) [4,5]; (b) grafting onto (GO, addition to a polymeric backbone of separately prepared side chains) [6]; and (c) grafting from (polymerization of side chains from a macroinitiator backbone) [7,8].

**Figure 1:**
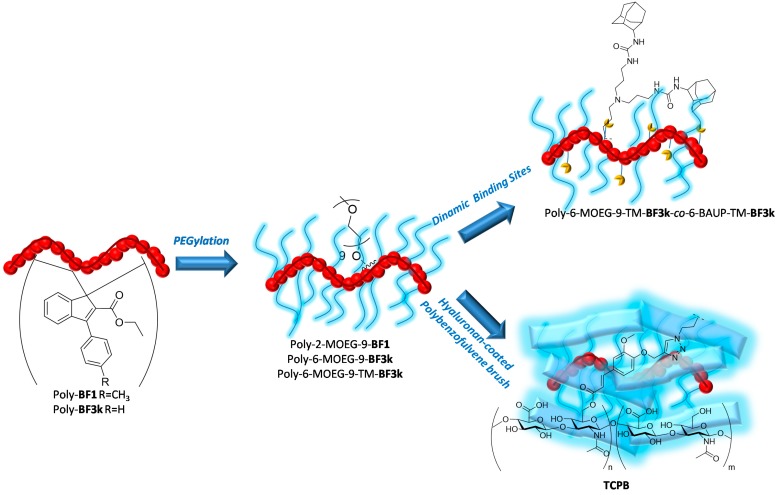
Development of PEGylated polybenzofulvene brushes (PPBFB) from poly-**BF1** and poly-**BF3k**.

A considerable number of vinyl monomers has been shown to undergo self-initiating spontaneous polymerization. Among them, our prototypical benzofulvene derivative **BF1** underwent spontaneous polymerization in the apparent absence of catalysts or initiators by evaporation of the solvent to deliver the corresponding polymer (i.e., poly-**BF1**, Figure 1). The characterization studies suggested, for this hydrophobic polybenzofulvene derivative, a vinyl structure stabilized by aromatic stacking interactions [9,10]. This intriguing finding led us to explore the chemistry of benzofulvene derivatives [11,12,13,14,15], and to prepare intriguing materials with potential applications in the optoelectronic field [16,17,18,19,20,21,22,23] and pharmaceutical science [24].

By combining different synthetic strategies, we designed and synthesized a large family of polymer brushes (Figure 1) obtained by grafting monomethyl oligo(ethylene glycol) (MOEG) side chains (SC) onto the polybenzofulvene backbone of poly-**BF1** and poly-**BF3k**. This new family of PEGylated polybenzofulvene brushes (PPBFB) includes poly-2-MOEG-9-**BF1** [25], poly-6-MOEG-9-**BF3k** [26], and poly-6-MOEG-9-TM-**BF3k** (TM = triazolo methyl spacer) [27,28].

Interestingly, PPBFB were capable of interacting with the water environment showing different behaviors from the generation of a compact physical hydrogel (poly-2-MOEG-9-**BF1**) [29] to the apparent water solubility of poly-6-MOEG-9-TM-**BF3k-GT** [27]. These results are suggestive of the crucial role played by the MOEG-9 SC, and their location in the monomeric unit in affecting aggregation features. Moreover, PPBFB were demonstrated to possess the suitable features to be considered good candidates for the development of nanostructured drug delivery systems. In particular, the physical hydrogel obtained with poly-2-MOEG-9-**BF1** was used in the delivery of a human immunoglobulin [29], whereas the thermoresponsive gel nanoparticles obtained with poly-6-MOEG-9-**BF3k** were used in the delivery the low molecular weight peptide anticancer drug leuprolide [30]. Very interestingly, the drug delivery systems obtained with these PPBFB derivatives appeared to show biocompatible and potentially bioresorbable features. Thus, in a higher level of macromolecular organization, the architecture of poly-6-MOEG-9-TM-**BF3k** was used in the development of a PPBFB copolymer (i.e., poly-6-MOEG-9-TM-**BF3k**-co-6-BAUP-TM-**BF3k**) bearing dynamic binding sites (BAUP = bis(adamantylurea) pincer) [31,32,33], capable of interacting with the anticancer drug doxorubicin [34]. This functional material was used to generate biocompatible gel nanoparticles showing dimensions around 200 nm, and capable of transporting and releasing the anticancer drug to tumor cells [34]. Finally, in the attempt of exploiting the potential targeting features of hyaluronic acid (HA), the tri-component polybenzofulvene brush (**TCPB**) material was developed as a third-generation PPBFB [35]. In this material, the core–shell architecture was further articulated by the presence of the external layer composed by the low molecular weight HA macromolecules. Thus, the biomimetic coat of **TCPB** material could interact with CD44 receptors, which are overexpressed in many tumor cells. **TCPB** material was used to prepare colloidal water dispersions containing nanoaggregates in the form of nanoparticles showing dimensions around 120 nm, that were devoid of cytotoxicity and capable of transporting and releasing the anticancer drug doxorubicin to tumor cells by exploiting active targeting based on the interaction of HA with CD44 receptors [36].

**Figure 2:**
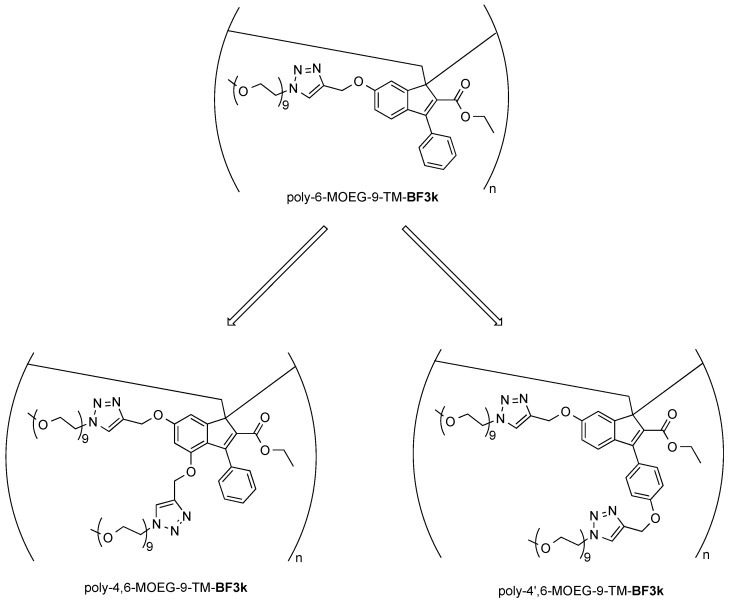
Design of PPBFB poly-4,6-MOEG-9-TM-**BF3k** and poly-4’,6-MOEG-9-TM-**BF3k** by the introduction of a second water-solubilizing MOEG-9 in the structure of poly-6-MOEG-9-TM-**BF3k** monomeric unit.

Here, in an attempt to modulate the aggregation features of these PEGylated polybenzofulvene brushes, a second water-solubilizing MOEG-9 side chain was introduced in the structure of poly-6-MOEG-9-TM-**BF3k** monomeric unit to obtain poly-4,6-MOEG-9-TM-**BF3k** and poly-4’,6-MOEG-9-TM-**BF3k** (Figure 2).

Thus as in a “grafting onto” approach, tailored benzofulvene monomers 4,6-PO-**BF3k** and 4’,6-PO-**BF3k** (see scheme in Section 3.1) bearing two propargyl groups were synthesized and induced to polymerize spontaneously by solvent removal to obtain the corresponding polybenzofulvene derivatives (i.e., poly-4,6-PO-**BF3k** and poly-4’,6-PO-**BF3k**) bearing clickable propargyl groups, which were exploited in the copper(I)-catalyzed alkyne-azide 1,3-dipolar cycloaddition (CuAAC, click chemistry reactions) [37] leading to poly-4,6-MOEG-9-TM-**BF3k** and poly-4’,6-MOEG-9-TM-**BF3k**, respectively, bearing a second MOEG-9 side chain with respect to the previously reported PPBFB. The presence of a lipophilic backbone bearing amphiphilic side chains generates two ideal compartments within the macromolecules. The first one, showing hydrophobic features, is potentially able to segregate small drug molecules, whereas the second one, showing amphiphilic features, is capable of interacting with the aqueous medium and promoting a progressive migration of the small drug molecules from the inside of the macromolecule toward the surrounding water environment. Interestingly, in the new polymers, the additional solubilizing moiety seems to be capable of generating substantial amounts of isolated macromolecules in water solution, circumventing the hydrophobic interaction leading to aggregation observed in the previously reported PPBFB. The aggregation properties in water solution of the novel densely PEGylated polybenzofulvene brushes were investigated by dynamic light scattering (DLS) and transmission electron microscopy (TEM) analysis. Moreover, owing to the double compartment (hydrophobic/amphiphilic) feature of this class of polymer brushes, preliminary cytotoxicity studies were performed in view of a potential application in drug encapsulation.

## 2. Materials and Methods

### 2.1. Synthesis

Melting points were determined in open capillaries in a Gallenkamp apparatus, and are uncorrected. Merck silica gel 60 (230–400 mesh) was used for column chromatography. Merck TLC plates, silica gel 60 F_254_, were used for TLC. Bruker (Karlsruhe, Germany) AC200, a Varian Mercury-300 (Palo Alto, CA, USA), a Bruker DRX-400 AVANCE, or a Bruker DRX-600 AVANCE spectrometer in the indicated solvents (TMS as internal standard) were used to record the NMR spectra. In NMR spectra, the coupling constants (*J*) in Hz and the values of the chemical shifts are expressed in ppm. Mass spectrometry experiments were performed using an Agilent (Santa Clara, CA, USA) 1100 LC/MSD operating with an electrospray source.

#### 2.1.1. Ethyl 2-(3,5-dihydroxybenzyl)-3-Oxo-3-Phenylpropanoate (**2**)

A mixture of ethyl benzoyl acetate (1.15 mL, 6.65 mmol) in DMF (10 mL) containing K_2_CO_3_ (2.76 g, 20 mmol), NaI (1.0 g, 6.67 mmol), and bromide **1** (1.35 g, 6.65 mmol), was stirred at room temperature for 2 h. The reaction mixture was then diluted with a saturated solution of ammonium chloride and extracted with ethyl acetate. The organic layer was dried over sodium sulfate and concentrated under reduced pressure. Purification of the residue by flash chromatography with petroleum ether/ethyl acetate (1:1) gave compound **2** as a white solid (1.94 g, yield 93%, mp 83–85 °C). ^1^H NMR (200 MHz, CDCl_3_): 1.09 (t, *J* = 7.0, 3H), 3.17 (d, *J* = 7.4, 2H), 4.08 (q, *J* = 7.1, 2H), 4.60 (t, *J* = 7.3, 1H), 6.20 (t, *J* = 1.8, 1H), 6.28 (d, *J* = 1.7, 2H), 7.43 (m, 2H), 7.54 (m, 1H), 7.92 (m, 2H). MS (ESI, negative ions): *m*/*z* 313 [M – H]^-^.

#### 2.1.2. Ethyl 4,6-Dihydroxy-3-Phenyl-1H-Indene-2-Carboxylate (**3**)

A mixture of compound **2** (2.2 g, 7.0 mmol) in polyphosphoric acid (PPA, 22 g) was mechanically stirred at room temperature for 30 min, and then cautiously decomposed with water. The white precipitate was extracted with ethyl acetate and the organic layer was dried over sodium sulfate and concentrated under reduced pressure. The residue was purified by flash chromatography with petroleum ether/ethyl acetate (1:1) as the eluent to obtain **3** as white crystals (1.3 g, yield 63%, mp 101–103 °C). ^1^H NMR (200 MHz, CDCl_3_): 1.02 (t, *J* = 7.0, 3H), 3.77 (s, 2H), 4.03 (q, *J* = 7.1, 2H), 5.00 (s, 1H), 5.28 (br s, 1H), 6.23 (s, 1H), 6.61 (s, 1H), 7.43 (m, 5H). MS (ESI): *m*/*z* 319 [M + Na]^+^.

#### 2.1.3. Ethyl 4,6-Dihydroxy-1-Oxo-3-Phenyl-1H-Indene-2-Carboxylate (**4**)

A solution of **3** (0.20 g, 0.675 mmol) in CH_2_Cl_2_ (20 mL) containing imidazole (0.32 g, 4.7 mmol) was cooled at 0–5 °C and TBDMSCl (0.51 g, 3.38 mmol) was added. The resulting mixture was stirred at 0–5 °C for 30 min, and at room temperature overnight. The reaction mixture was partitioned between dichloromethane and water, and the organic layer was dried over sodium sulfate and concentrated under reduced pressure. A mixture of the resulting residue in 1,4-dioxane (10 mL) with SeO_2_ (0.21 g, 1.89 mmol) was heated to reflux overnight, and then treated with a saturated solution of NaHCO_3_. The red mixture was extracted with diethyl ether and the organic layer was dried over sodium sulfate and concentrated under reduced pressure to afford a red residue, which was dissolved in THF (15 mL) and treated with a 1 M tetrabutylammonium fluoride solution (2.0 mL, 2.0 mmol). After stirring at room temperature for 40 min, water was added, and the mixture was concentrated under reduced pressure. The residue was partitioned between water and ethyl acetate, and the organic layer was washed with water, dried over sodium sulfate, and concentrated under reduced pressure. Purification of the residue by flash chromatography with ethyl acetate as the eluent gave indenone derivative **4** as a red solid (0.12 g, yield 57%, mp 195–196 °C). ^1^H NMR (200 MHz, CD_3_OD): 0.94 (t, *J* = 7.0, 3H), 3.95 (q, *J* = 7.1, 2H), 6.21 (d, *J* = 1.8, 1H), 6.55 (d, *J* = 1.6, 1H), 7.38 (m, 5H). MS (ESI): *m*/*z* 333 [M + Na]^+^.

#### 2.1.4. Ethyl 1-Oxo-3-Phenyl-4,6-Bis(prop-2-ynyloxy)-1H-Indene-2-Carboxylate (**5**)

A mixture of **4** (0.50 g, 1.61 mmol) in acetone (20 mL) containing K_2_CO_3_ (1.34 g, 9.70 mmol), NaI (0.72 g, 4.80 mmol) and propargyl bromide (0.047 mL, 5.31 mmol) was refluxed for 5 h. The reaction mixture was then quenched with water, and extracted with ethyl acetate. The organic layer was dried over sodium sulfate and concentrated under reduced pressure. The residue was purified by flash chromatography with petroleum ether/ethyl acetate (8:2) as the eluent to afford **5** as a red solid (0.25 g, yield 40%). An analytical sample was obtained by recrystallization from ethyl acetate by slow evaporation of the solvent to obtain red crystals melting at 122.5–123 °C. ^1^H NMR (200 MHz, CDCl_3_): 1.03 (t, *J* = 7.0, 3H), 2.45 (t, *J* = 2.3, 1H), 2.56 (t, *J* = 1.7, 1H), 4.07 (q, *J* = 7.1, 2H), 4.37 (d, *J* = 1.9, 2H), 4.73 (d, *J* = 2.5, 2H), 6.63 (d, *J* = 1.8, 1H), 6.94 (d, *J* = 1.5, 1H), 7.39 (m, 5H).

#### 2.1.5. Ethyl 1-Hydroxy-1-Methyl-3-Phenyl-4,6-Bis(prop-2-ynyloxy)-1H-Indene-2-Carboxylate (**6**)

To a solution of indenone, derivative **5** (0.39 g, 1.01 mmol) in dichloromethane (30 mL) was added a 2M solution of Al(CH_3_)_3_ in THF (1.0 mL, 2.0 mmol). The reaction mixture was stirred under a nitrogen atmosphere at room temperature for 30 min, and then diluted with ethyl acetate (20 mL). The Al(CH_3_)_3_ excess was cautiously destroyed with a 1 M NaOH solution (2.0 mL), and the resulting mixture was partitioned between water and ethyl acetate. The organic layer was dried over sodium sulfate and concentrated under reduced pressure. Purification of the residue by flash chromatography with petroleum ether/ethyl acetate (8:2) as the eluent gave indenol **6** as a white solid (0.28 g, yield 69%, mp 88–89 °C). ^1^H NMR (200 MHz, CDCl_3_): 0.92 (t, *J* = 7.2, 3H), 1.71 (s, 3H), 2.37 (t, *J* = 2.3, 1H), 2.56 (t, *J* = 1.7, 1H), 3.78 (br s, 1H), 4.01 (m, 2H), 4.26 (d, *J* = 2.0, 2H), 4.73 (d, *J* = 2.0, 2H), 6.52 (d, *J* = 2.1, 1H), 6.87 (d, *J* = 1.8, 1H), 7.29 (m, 5H). MS (ESI): *m*/*z* 425 [M + Na]^+^.

#### 2.1.6. Ethyl 1-Methylene-3-Phenyl-4,6-Bis(prop-2-ynyloxy)-1H-Indene-2-Carboxylate (4,6-PO-**BF3k**)

A mixture of indenol derivative **6** (0.30 g, 0.745 mmol) in chloroform or CDCl_3_ (25 mL) with a catalytic amount of *p*-toluenesulfonic acid monohydrate (PTSA, 0.028 g, 0.147 mmol) was heated at 50 °C for 10–15 min, and cooled to room temperature. The reaction mixture was then washed with a saturated solution of NaHCO_3_, and dried over sodium sulfate to afford a solution of crude monomer 4,6-PO-**BF3k**, which was used for the preparation of poly-4,6-PO-**BF3k**. An analytical sample of the monomer was obtained by flash chromatography purification (CDCl_3_ as the eluent) of 4,6-PO-**BF3k** solution without the final evaporation of the eluent. ^1^H NMR (400 MHz, CDCl_3_): 0.93 (t, *J* = 7.2, 3H), 2.38 (t, *J* = 2.4, 1H), 2.55 (t, *J* = 2.4, 1H), 4.01 (q, *J* = 7.2, 2H), 4.30 (d, *J* = 2.4, 2H), 4.75 (d, *J* = 2.4, 2H), 6.33 (s, 1H), 6.57 (d, *J* = 2.0, 1H), 6.60 (s, 1H), 7.03 (d, *J* = 2.0, 1H), 7.32 (m, 5H). MS (ESI): *m*/*z* 407 [M + Na]^+^.

#### 2.1.7. Poly[ethyl 1-methylene-3-phenyl-4,6-bis(prop-2-ynyloxy)-1H-indene-2-carboxylate] (Poly-4,6-PO-**BF3k**)

A chloroform solution of crude monomer 4,6-PO-**BF3k** was concentrated under reduced pressure. The residue was dissolved in chloroform (10 mL) and newly concentrated (the procedure of dissolution/evaporation was applied three times). The final residue was dissolved in the minimum amount of chloroform and precipitated with methanol. The obtained powder was dried under reduced pressure to obtain poly-4,6-PO-**BF3k** as a white solid (0.11 g, yield 38%). ^1^H NMR (600 MHz, CDCl_3_): see figures in Section 3.2.1. ^13^C NMR (75 MHz, CDCl_3_): see figures in Section 3.2.1.

#### 2.1.8. Poly[ethyl 4,6-bis[[1-(2,5,8,11,14,17,20,23,26-nonaoxaoctacosan-28-yl)-1H-1,2,3-triazol-4-yl]methoxy]-1-methylene-3-phenyl-1H-indene-2-carboxylate] (Poly-4,6-MOEG-9-TM-**BF3k**)

A mixture of poly-4,6-PO-**BF3k** (50 mg, 0.13 mmol in monomeric unit) in THF (3.0 mL) containing 28-azido-2,5,8,11,14,17,20,23,26-nonaoxaoctacosane [27] (0.18 g, 0.397 mmol), CuBr (4.0 mg, 0.028 mmol), and *N,N*-Diisopropylethylamine (DIPEA) (0.005 mL, 0.029 mmol) was submitted to microwave irradiation into a CEM (Matthews, NC, USA) Discover apparatus (6 cycles of 10 min at 150 W and 60 °C). The solvent was then removed under reduced pressure, and the residue was treated with water (15 mL) at room temperature. After the addition of a 33% ammonia solution (3.0 mL), the mixture was stirred at room temperature overnight, and then partitioned between chloroform and brine. The aqueous phase was extracted with chloroform and the combined organic extracts were dried over sodium sulfate and concentrated under reduced pressure. The resulting residue was purified by washing with *n*-hexane to obtain poly-**2b** (0.080 g, yield 48%) as a yellow sticky solid. ^1^H NMR (600 MHz, CDCl_3_): see figure in Section 3.3.1. ^13^C NMR (75 MHz, CDCl_3_): see figure in Section 3.3.1.

#### 2.1.9. Ethyl 6-Hydroxy-3-(4-hydroxyphenyl)-1-Oxo-1H-Indene-2-Carboxylate (**8**)

To a solution of indenone derivative **7** [15] (0.70 g, 2.07 mmol) in dichloromethane (20 mL) cooled to 0 °C, was added, dropwise, a solution (1 M in dichloromethane) of BBr_3_ (16.6 mL, 16.6 mmol), and the reaction mixture was stirred overnight at room temperature. A saturated NaHCO_3_ solution was then added until the gas evolution ceased. The resulting mixture was extracted with diethyl ether, and the organic layer was dried over sodium sulfate and evaporated under reduced pressure. The residue was purified by flash chromatography with petroleum ether/ethyl acetate (9:1) as the eluent to obtain pure compound **8** as a dark red solid (0.55 g, yield 86%, mp 224–225 °C). ^1^H NMR (400 MHz, CD_3_OD): 1.14 (t, *J* = 7.1, 3H), 4.14 (q, *J* = 7.1, 2H), 6.79 (dd, *J* = 8.1, 2.3, 1H), 6.91 (d, *J* = 8.6, 2H), 6.95 (d, *J* = 2.3, 1H), 7.15 (d, *J* = 8.1, 1H), 7.43 (d, *J* = 8.6, 2H). MS (ESI): *m*/*z* 333 [M + Na]^+^.

#### 2.1.10. Ethyl 1-Oxo-6-(prop-2-ynyloxy)-3-[4-(prop-2-ynyloxy)phenyl)-1H-Indene-2-Carboxylate (**9**)

A mixture of **8** (0.50 g, 1.61 mmol) in acetone (20 mL) containing Cs_2_CO_3_ (3.15 g, 9.67 mmol), NaI (0.72 g, 4.80 mmol), and propargyl bromide (0.047 mL, 5.31 mmol) was refluxed for 5 h. The reaction mixture was then quenched with water, acidified with 6 N HCl, and extracted with diethyl ether. The organic layer was dried over sodium sulfate, and concentrated under reduced pressure. The residue was purified by flash chromatography with petroleum ether/ethyl acetate (8:2) as the eluent, to afford **9** as an orange solid (0.31 g, yield 50%). An analytical sample was obtained by recrystallization from diethyl ether by slow evaporation of the solvent to obtain red-orange crystals melting at 127.5–128 °C. ^1^H NMR (400 MHz, CDCl_3_): 1.20 (t, *J* = 7.1, 3H), 2.54–2.56 (m, 2H), 4.20 (q, *J* = 7.1, 2H), 4.73 (d, *J* = 2.1, 2H), 4.76 (d, *J* = 2.1, 2H), 6.93 (dd, *J* = 8.1, 2.2, 1H), 7.08 (d, *J* = 8.6, 2H), 7.16 (d, *J* = 8.1, 1H), 7.22 (d, *J* = 2.2, 1H), 7.53 (d, *J* = 8.6, 2H). MS (ESI): *m*/*z* 409 [M + Na]^+^.

#### 2.1.11. Ethyl 1-Hydroxy-1-Methyl-6-(prop-2-ynyloxy)-3-[4-(prop-2-ynyloxy)phenyl)-1H-Indene -2-Carboxylate (**10**)

To a solution of indenone derivative **9** (0.49 g, 1.27 mmol) in dichloromethane (20 mL), was added a 2 M solution of Al(CH_3_)_3_ in THF (1.27 mL, 2.54 mmol). The reaction mixture was stirred under a nitrogen atmosphere at room temperature for 15 min, and then diluted with ethyl acetate (20 mL). The Al(CH_3_)_3_ excess was cautiously destroyed with a 1 M NaOH solution (2.0 mL), and the resulting mixture was partitioned between water and ethyl acetate. The organic layer was dried over sodium sulfate and concentrated under reduced pressure. Purification of the residue by flash chromatography with petroleum ether/ethyl acetate (9:1) as the eluent afforded indenol derivative **10** as a pale yellow glassy solid (0.48 g, yield 94%). ^1^H NMR (400 MHz, CDCl_3_): 1.09 (t, *J* = 7.1, 3H), 1.74 (s, 3H), 2.52–2.54 (m, 2H), 3.59 (br s, 1H), 4.10–4.12 (m, 2H), 4.72 (m, 4H), 6.87 (dd, *J* = 8.4, 1.9, 1H), 7.02 (d, *J* = 8.6, 2H), 7.10 (d, *J* = 8.4, 1H), 7.19 (d, *J* = 1.9, 1H), 7.34 (d, *J* = 8.6, 2H). MS (ESI): *m*/*z* 425 [M + Na]^+^.

#### 2.1.12. Ethyl 1-Methylene-6-(prop-2-ynyloxy)-3-[4-(prop-2-ynyloxy)phenyl)-1H-Indene-2-Carboxylate (4’,6-PO-**BF3k**)

A mixture of indenol derivative **10** (0.36 g, 0.89 mmol) in chloroform or CDCl_3_ (18 mL) with a catalytic amount of *p*-toluenesulfonic acid monohydrate (PTSA, 0.028 g, 0.15 mmol) was refluxed for 1 h, and then cooled to room temperature. The reaction mixture was washed with a saturated solution of NaHCO_3_ and dried over sodium sulfate to afford a solution (around 0.05 M) of monomer 4’,6-PO-**BF3k**, which was used for the preparation of poly-4’,6-PO-**BF3k**. ^1^H NMR (400 MHz, CDCl_3_): 1.10 (t, *J* = 7.1, 3H), 2.52–2.54 (m, 2H), 4.14 (q, *J* = 7.1, 2H), 4.73 (m, 4H), 6.32 (s, 1H), 6.58 (s, 1H), 6.91 (dd, *J* = 8.4, 2.3, 1H), 7.04 (d, *J* = 8.7, 2H), 7.17 (d, *J* = 8.4, 1H), 7.32 (d, *J* = 2.2, 1H), 7.37 (d, *J* = 8.7, 2H). MS (ESI): *m*/*z* 407 [M + Na]^+^.

#### 2.1.13. Poly[ethyl 1-methylene-6-(prop-2-ynyloxy)-3-[4-(prop-2-ynyloxy)phenyl)-1H-indene-2-carboxylate] (Poly-4’,6-PO-**BF3k**)

A solution of monomer 4’,6-PO-**BF3k** in chloroform was concentrated under reduced pressure to give a yellow viscous oil, which was dissolved in chloroform (10 mL) and newly evaporated (this procedure of dissolution/evaporation was repeated 8 times). The final residue was purified by precipitation with methanol from a solution of the polymer in chloroform, and dried under reduced pressure to obtain poly-4’,6-PO-**BF3k** as a white solid (0.26 g, yield 76%). ^1^H NMR (600 MHz, CDCl_3_): see figures in Section 3.2.1. ^13^C NMR (150 MHz, CDCl_3_): see figures in Section 3.2.1.

#### 2.1.14. Poly[ethyl 1-methylene-6-[[1-(2,5,8,11,14,17,20,23,26-nonaoxaoctacosan-28-yl)-1H-1,2,3-triazol-4-yl]methoxy]-3-[4-[[1-(2,5,8,11,14,17,20,23,26-nonaoxaoctacosan-28-yl)-1H-1,2,3-triazol-4-yl]methoxy]phenyl]-1H-indene-2-carboxylate] (Poly-4’,6-MOEG-9-TM-**BF3k**)

A mixture of poly-4’,6-PO-**BF3k** (50 mg, 0.13 mmol in monomeric unit) in DMF (5.0 mL) containing 28-azido-2,5,8,11,14,17,20,23,26-nonaoxaoctacosane [27] (240 mg, 0.529 mmol), CuBr (4.0 mg, 0.028 mmol), and DIPEA (0.005 mL, 0.029 mmol) was submitted to microwave irradiation into a CEM Discover apparatus (4 cycles of 10 min at 150 W and 60 °C). The solvent was then removed under reduced pressure, and the residue was treated with water (15 mL) at room temperature. After the addition of a 33% ammonia solution (3.0 mL), the mixture was stirred at room temperature overnight, and then partitioned between chloroform and brine. The aqueous phase was extracted with chloroform, and the combined organic extracts were dried over sodium sulfate and concentrated under reduced pressure. The resulting residue was dissolved into THF, and then treated with QUADRASIL MP (500 mg). After filtration, the solution was concentrated under reduced pressure, and the final residue was purified by precipitation with *n*-hexane from a solution of the polymer in chloroform (the precipitation procedure was repeated two times), and dried under reduced pressure to obtain poly-4’,6-MOEG-9-TM-**BF3k** as a yellow sticky solid (90 mg, yield 54%). ^1^H NMR (300 MHz, CDCl_3_): see figure in Section 3.3.1. ^13^C NMR (75 MHz, CDCl_3_): see figure in Section 3.3.1.

### 2.2. X-ray Crystallography

An Oxford Diffraction Xcalibur Sapphire 3 diffractometer (Santa Clara, CA, USA) with a graphite monochromated Mo-Kα radiation (λ = 0.71073 Å) at 293 K was used to collect X-ray data with the single crystals of **5** and **9**. The structures were solved by direct methods implemented in SHELXS-2013 program [38]. The refinements were carried out by full-matrix anisotropic least-squares on F2 for all reflections for non-H atoms by means of the SHELXL-2018 program [39]. The crystallographic data (excluding structure factors) have been deposited with the Cambridge Crystallographic Data Centre as supplementary publications no. CCDC 1868493 (**5**) and 1868494 (**9**). Copies of the data can be obtained, free of charge, on application to CCDC, 12 Union Road, Cambridge CB2 1EZ, UK.

### 2.3. Mass Spectrometry

MALDI TOF MS (matrix assisted laser desorption/ionization time of flight mass spectrometry) mass spectra of the polymers were recorded in linear delayed extraction mode using a Voyager-DE STR (PerSeptive Biosystems, Foster city, CA, USA) mass spectrometer, equipped with a nitrogen laser emitting at 337 nm, with a 3 ns pulse width, and working in positive ion mode. The accelerating voltage was 20 kV, and the grid voltage and delay time (delayed extraction, time lag) were optimized for each sample to achieve the highest mass resolution, expressed as the molar mass of a given ion divided by the full width at half-maximum (fwhm). The laser irradiance was maintained slightly above the threshold. A resolution of 800–1000 was obtained in the mass range 800–10,000. External calibration was performed using a homemade low-molar-mass (*M*_n_ = 6000 g/mol) hydroxy-ended poly(bisphenol A carbonate). Mass accuracy was about 150–250 ppm in the mass range 1000–3500 Da, and 400 ppm in the mass range 3500–9000 Da. Several matrices, such as trans-2-[3-(4-tert-butylphenyl)-2-methyl-2-propenylidene]malononitrile (DCTB), 2-(4-hydroxyphenylazo)-benzoic acid (HABA), 1,8,9-anthracenetriol (dithranol), and α-cyano-4-hydroxycinnamic acid (referred as α-CHCA), were used. All matrices were solubilized in the appropriate solvent (THF, CHCl_3_, DMF) at a concentration of 0.1 M, polymer samples were solubilized in the specific good solvent (i.e., CHCL_3_ for the poly-4’,6-PO-**BF3k** and poly-4,6-PO-**BF3k** sample; DMF for the poly-4,6-MOEG-9-TM-**BF3k** and poly-4’,6-MOEG-9-TM-**BF3k**) with a concentration of about 2 mg/mL.

Generally, 0.5 μL of a polymer solution was mixed with 0.5 μL of a matrix solution, and then spotted on the MALDI sample holder, and slowly dried to allow matrix crystallization. The best spectra were recorded with the matrix: α-CHCA for poly-4,6-PO-**BF3k**; HABA for poly-4’,6-PO-**BF3k**; DCTB both for poly-4,6-MOEG-9-TM-**BF3k** and poly-4’,6-MOEG-9-TM-**BF3k**.

### 2.4. Wide-Angle X-ray Diffraction Studies

Wide-angle X-ray diffraction (WAXD) data were obtained at 20 °C using a Siemens D-500 diffractometer (Bruker AXS, Inc., Madison, WI, USA) equipped with a Siemens FK 60–10 2000W tube (the radiation was a monochromatized Cu K_α_ beam with wavelenght *λ* = 0.154 nm). The operating voltage and current were 40 kV and 40 mA, respectively. The data were collected from 2 to 40 2θ° at 0.02 2θ° intervals.

### 2.5. SEC-MALS

The MWD (molecular weight distribution) characterization of the polybenzofulvene derivatives was performed using a Wyatt MALS (Santa Barbara, CA, USA) light scattering photometer on-line to a Waters SEC chromatographic system (Milford, MA, USA). The SEC-MALS apparatus and the corresponding experimental conditions were the same used in previous analysis [11,25,28].

### 2.6. Dynamic Light Scattering (DLS) Analysis and ζ Potential Measurements

A Zetasizer Nano ZS (Worcestershire, UK) instrument fitted with a 532 nm laser at a fixed scattering angle of 173° was used for the determination of the mean diameter, width of distribution (polydispersity index, PDI), and ζ potential of the nanoparticles at 25 °C. The intensity-average hydrodynamic diameter (size in nm) and PDI of nanosystems were measured in double distilled water after 2 and 20 days. Polymer dispersions were prepared at room temperature by stirring samples (0.2–2 mg/mL) until complete dispersion (2 days). In order to calculate the Z-potential (mV), Smoluchowsky relationship was applied assuming that K·*a* >> 1 (where K is the Debye-Hückel parameter and a is particle radius). Experiments were carried out in triplicate.

### 2.7. Transmission Electron Microscopy (TEM)

A drop of 3.5 μL of poly-4’,6-MOEG-9-TM-**BF3k** solution in water was dropped onto a 300 mesh formvar-coated copper grid. After 2 min, the excess of sample was blotted by filter paper and the grids were stained with 1% of aqueous uranyl acetate solution. Samples were observed in a FEI Tecnai G2 Spirit transmission electron microscopy (Hillsboro, OR, USA) at an acceleration voltage of 100 kV.

### 2.8. Cytotoxicity Evaluation

The biocompatibility of polybenzofulvene derivatives was evaluated on human colon cancer (HCT116) by tetrazolium salt (MTS) assay (Promega, Madison, WI, USA). In this regard, cells were seeded in 96-well plates at density of 1 × 10^4^ cells/well and grown for 24 h in supplemented Dulbecco’s Minimum Essential Medium (DMEM). After that, cells were incubated with 200 μL of DMEM containing poly-4,6-MOEG-9-TM-**BF3k** or poly-4’,6-MOEG-9-TM-**BF3k** at concentrations per well ranging from 2 to 0.1 mg/mL for 24 and 48 h. Cells incubated with fresh culture medium were used as negative control. After the incubation time, the dispersion of polybenzofulvene derivatives was removed, and cells were treated with a 120 μL of a solution containing fresh medium and MTS solution (5:1). The absorbance at 490 nm was measured using a microplate reader (Multiskan, Thermo, UK) after 2 h of incubation. The viability was expressed as the percentage obtained from the ratio between each sample with respect to their negative control (100% of cell viability). All culture experiments were performed in triplicates.

### 2.9. Statistical Analysis

Data of three independent experiments are presented as average ± standard deviation for each treatment group in each experiment. Data were analyzed by one-way ANOVA. Statistical significance was set at *p* < 0.05.

## 3. Results and Discussion

### 3.1. Synthesis

**Scheme 1:**
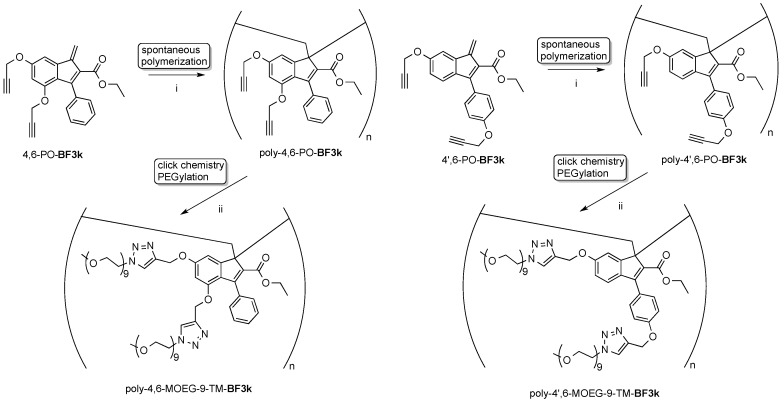
“Grafting onto” approach to PPBFB poly-4,6-MOEG-9-TM-**BF3k** (**left**) and poly-4’,6-MOEG-9-TM-**BF3k** (**right**). Reagents: (i) solvent elimination; (ii) CH_3_(OCH_2_CH_2_)_9_N_3_, CuBr, DIPEA, THF (or DMF), MW.

The “grafting onto” approach to PPBFB poly-4,6-MOEG-9-TM-**BF3k** and poly-4’,6-MOEG-9-TM-**BF3k** was depicted in Scheme 1. In particular, the two benzofulvene monomers 4,6-PO-**BF3k** and 4’,6-PO-**BF3k** bearing two propargyl groups were induced to polymerize spontaneously by solvent removal to obtain polybenzofulvene derivatives poly-4,6-PO-**BF3k** and poly-4’,6-PO-**BF3k**, which were thoroughly characterized by SEC-MALS, NMR spectroscopy, and MALDI-TOF MS techniques (see below). Then, the clickable propargyl groups of these two polybenzofulvene derivatives were exploited in CuAAC coupling with MOEG-9 chains bearing an azide group [27] in the presence of CuBr and DIPEA to obtain poly-4,6-MOEG-9-TM-**BF3k** and poly-4’,6-MOEG-9-TM-**BF3k**.

Since polybenzofulvene derivatives are generally liable to depolymerize when heated in the presence of solvents, the optimization of the conditions of the click chemistry (CuAAC) reaction required a particular care and, therefore, was guided by the experience acquired in the synthesis poly-6-MOEG-9-TM-**BF3k** [28]. Thus, in the simple conditions reported above, CuAAC reaction was confirmed to be almost exhaustive by microwave exposition without significant depolymerization.

**Scheme 2:**
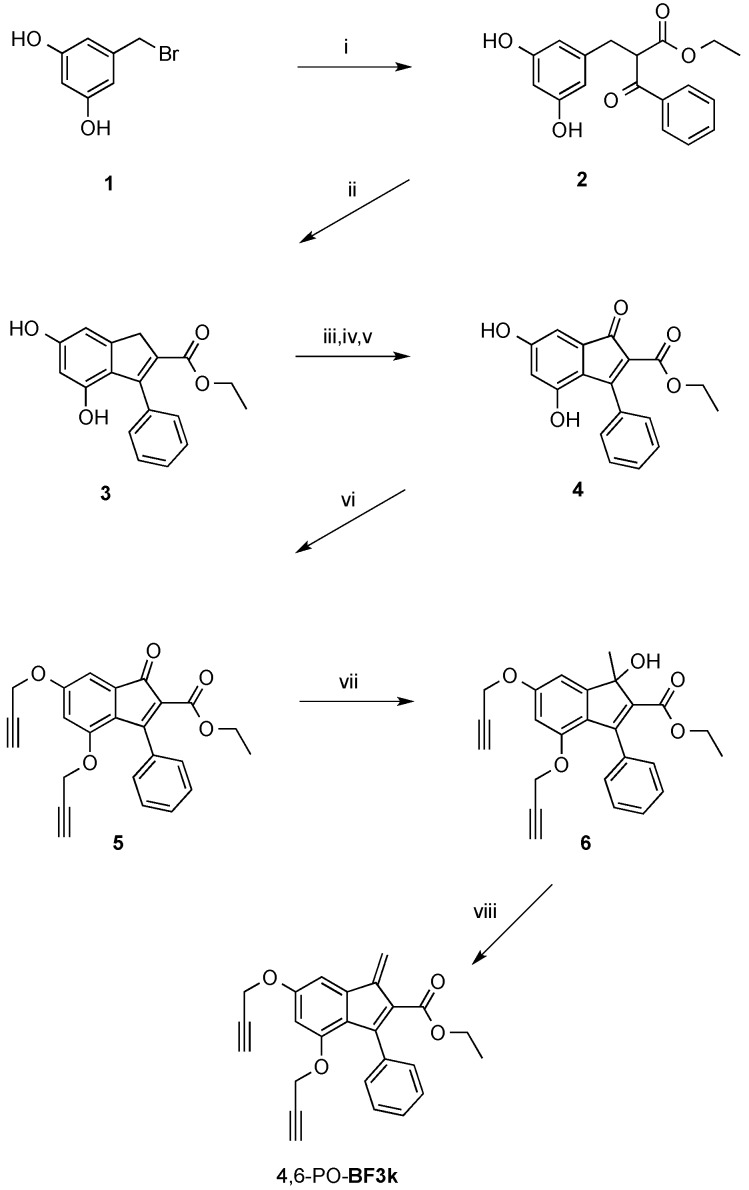
Synthesis of benzofulvene monomer 4,6-PO-**BF3k**. Reagents: (i) ethyl benzoylacetate, NaI, K_2_CO_3_, DMF; (ii) PPA; (iii) TBDMSCl, imidazole, CH_2_Cl_2_; (iv) SeO_2_, dioxane; (v) (C_4_H_9_)_4_NF, THF; (vi) propargyl bromide, NaI, K_2_CO_3_, CH_3_COCH_3_; (vii) Al(CH_3_)_3_, CH_2_Cl_2_; (viii) PTSA, CHCl_3_.

Benzofulvene monomer 4,6-PO-**BF3k** was prepared as described in Scheme 2.

**Figure 3:**
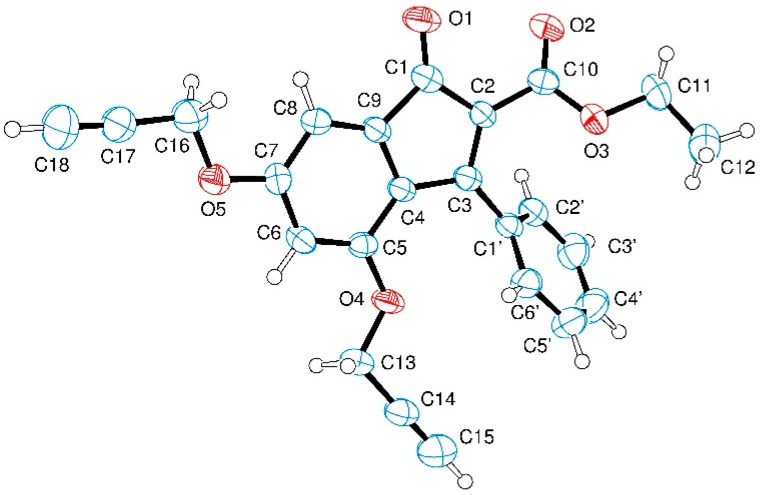
Structure of indenone derivative **5** found by crystallography. Ellipsoids enclose 50% probability.

Alkylation of ethyl benzoylacetate with bromide **1** [40] gave intermediate **2**, which was cyclized with polyphosphoric acid to give indene derivative **3**. Direct oxidation of **3** with selenium dioxide gave unsatisfactory results. Thus, **3** was submitted to a multistep protection–oxidation–deprotection procedure to obtain the expected indenone derivative **4**, which was reacted with propargyl bromide to obtain compound **5**. The structure of this indenone intermediate was confirmed by crystallographic studies (Figure 3).

Alkylation of **5** with trimethylaluminum gave the expected indenol derivative **6**, which was dehydrated in the presence of *p*-toluenesulfonic acid (PTSA) in the attempt to obtain monomer 4,6-PO-**BF3k**. In agreement with the results obtained with closely related compounds [13], the formation of this benzofulvene monomer was accompanied by the formation of several byproducts, as suggested by NMR studies of the reaction mixture. However, the solvent evaporation led to a rapid and exhaustive polymerization of benzofulvene monomer 4,6-PO-**BF3k** to give the corresponding polymer in the apparent absence of initiators or catalysts. The rapid spontaneous polymerization of this benzofulvene monomer in the complex reaction mixture suggests that the recognition process, which we assumed to be the initial step of the “affinity polymerization” pathway [14,15,27], is capable of operating in a selective and efficient way.

**Scheme 3:**
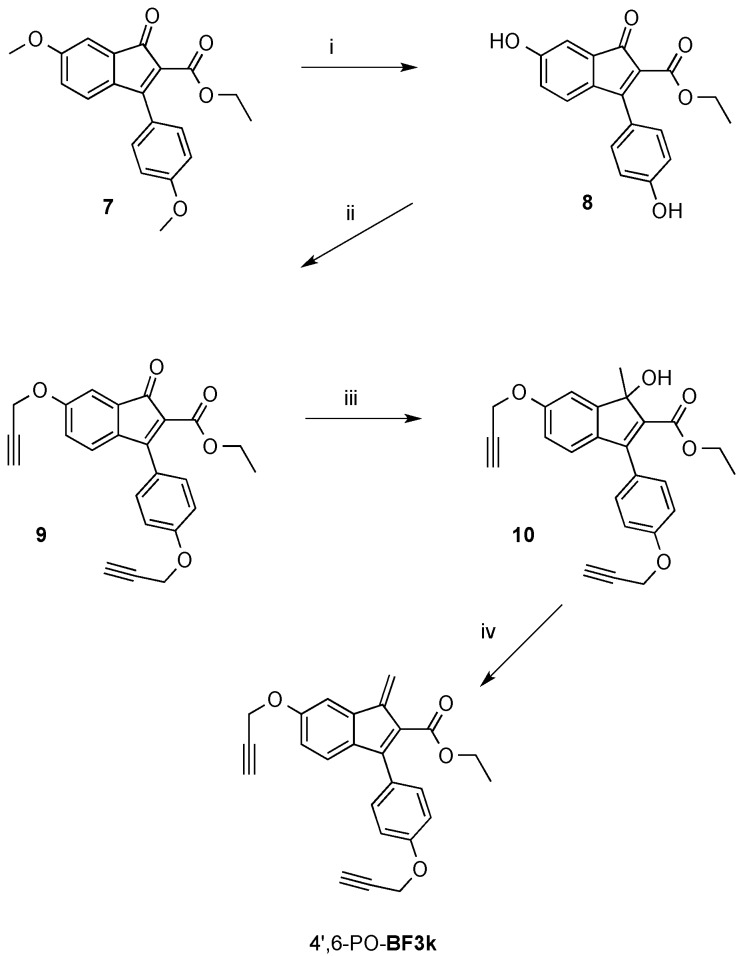
Synthesis of benzofulvene monomer 4’,6-PO-BF3k. Reagents: (i) BBr_3_, CH_2_Cl_2_; (ii) propargyl bromide, NaI, Cs_2_CO_3_, CH_3_COCH_3_; (iii) Al(CH_3_)_3_, CH_2_Cl_2_; (iv) PTSA, CHCl_3_.

On the other hand, benzofulvene monomer 4’,6-PO-**BF3k** was synthesized by applying the chemistry shown above to indenone derivative **7** (Scheme 3).

**Figure 4:**
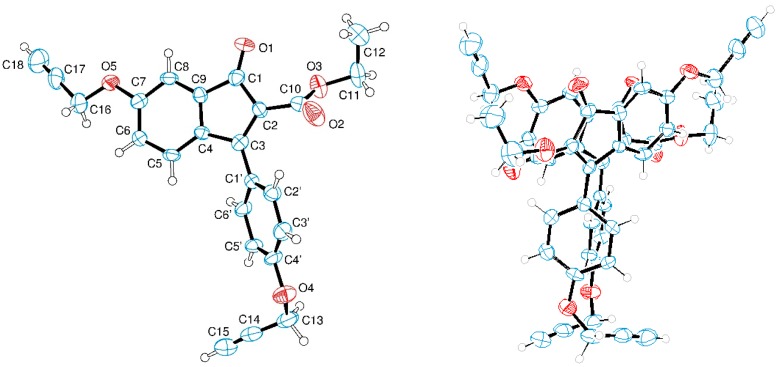
Structure of indenone derivative **9** found by crystallography. Single molecule (**left**) and two packed molecules (**right**). Ellipsoids enclose 50% probability.

Indenone **7** [15] was demethylated with BBr_3_ to afford compound **8**, which was, in turn, alkylated with propargyl bromide to obtain dipropargyloxy derivative **9**. The structure of this indenone intermediate was confirmed by crystallographic studies (Figure 4). Interestingly, the analysis of the crystal packing of this indenone derivative showed the presence of an aromatic T-shaped interaction among the pendant phenyl groups that has been suggested to play a crucial role in the recognition process, which we assumed to be the initial step of the “affinity polymerization” pathway [14,15,27].

Alkylation of **9** with trimethylaluminum gave the expected indenol derivative **6**, which was cleanly dehydrated in the presence of *p*-toluenesulfonic acid (PTSA) in to obtain pure monomer 4’,6-PO-**BF3k**.

### 3.2. Structure of Poly-4,6-PO-**BF3k** and Poly-4’,6-PO-**BF3k**

#### 3.2.1. NMR Spectroscopy

**Figure 5:**
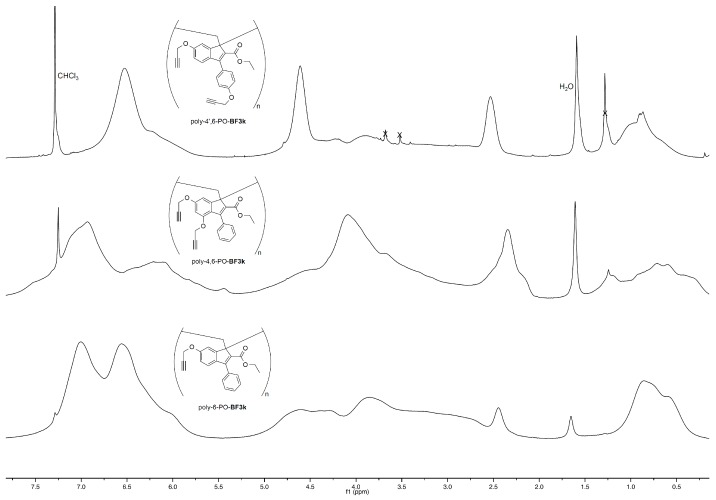
^1^H NMR spectra (600 MHz, CDCl_3_) of poly-4’,6-PO-**BF3k** (**top trace**) and poly-4,6-PO-**BF3k** (**middle trace**) compared with that of previously reported poly-6-PO-**BF3k** (**bottom trace**).

The ^1^H NMR spectra of poly-4,6-PO-**BF3k** and poly-4’,6-PO-**BF3k** (Figure 5) were relatively similar to that of the corresponding polybenzofulvene derivative poly-6-PO-**BF3k** bearing one propargyloxy group each monomeric unit with significant differences in the intensity of the signals of the propargyloxy groups at around 2.3–2.5 ppm. Moreover, significant differences were observed also in the chemical shift values of the signals attributed to the aromatic protons (because of the differences in the number and/or position of the electron-donating propargyloxy groups) and to the methylene group of the propargyloxy moieties.

**Figure 6:**
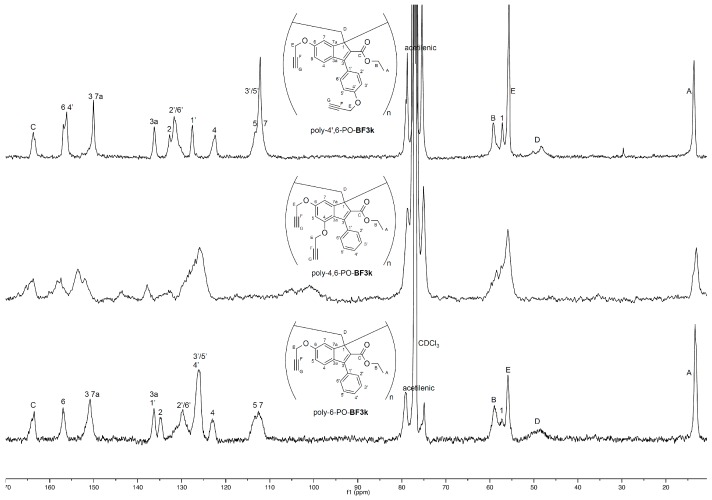
^13^C NMR spectra (CDCl_3_) of poly-4’,6-PO-**BF3k** (**top trace**) and poly-4,6-PO-**BF3k** (**middle trace**) compared with that of previously reported poly-6-PO-**BF3k** (**bottom trace**).

These differences were reflected also in the ^13^C NMR spectra shown in Figure 6.

In fact, the signals (at around 75 and 79 ppm) attributed to the carbon atoms of acetylene moieties showed higher intensities in the spectra of the newly synthesized polybenzofulvene derivatives of poly-4,6-PO-**BF3k** and poly-4’,6-PO-**BF3k**, with respect to the previously described poly-6-PO-**BF3k**.

**Figure 7:**
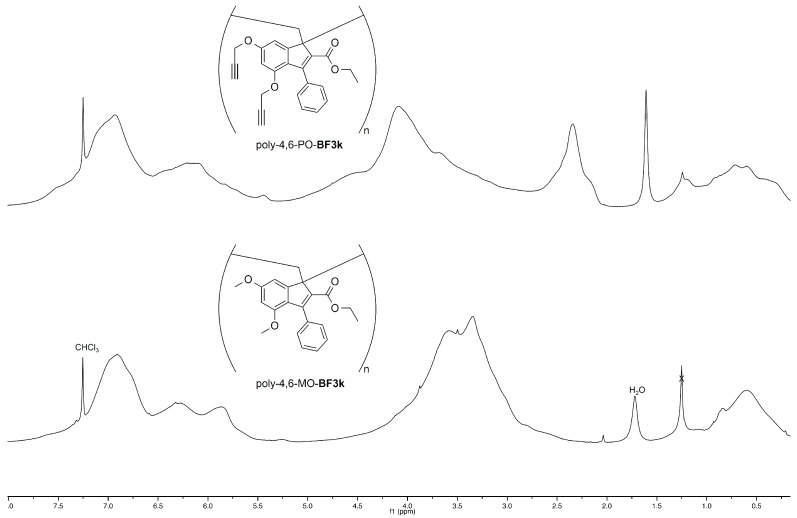
^1^H NMR spectrum (CDCl_3_) of poly-4,6-PO-**BF3k** (**top trace**) compared with that of previously reported poly-4,6-MO-**BF3k** (**bottom trace**).

**Figure 8:**
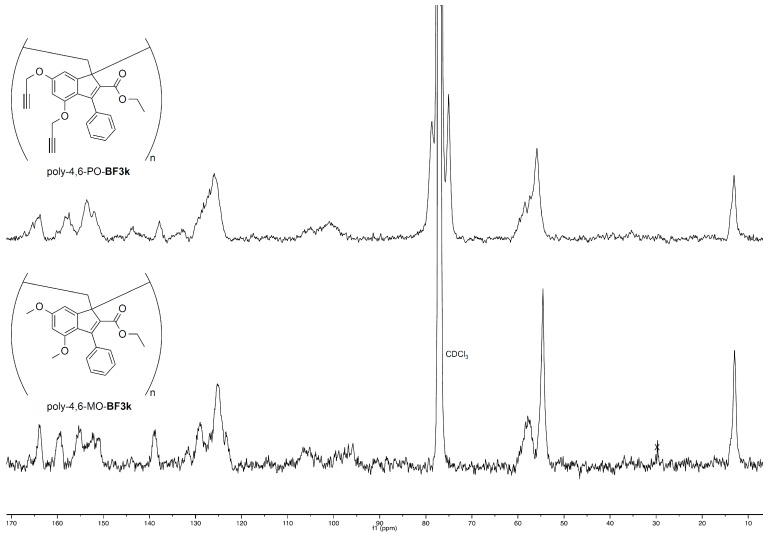
^13^C NMR spectrum (CDCl_3_) of poly-4,6-PO-**BF3k** (top trace) compared with that of previously reported poly-4,6-MO-**BF3k** (bottom trace).

**Figure 9:**
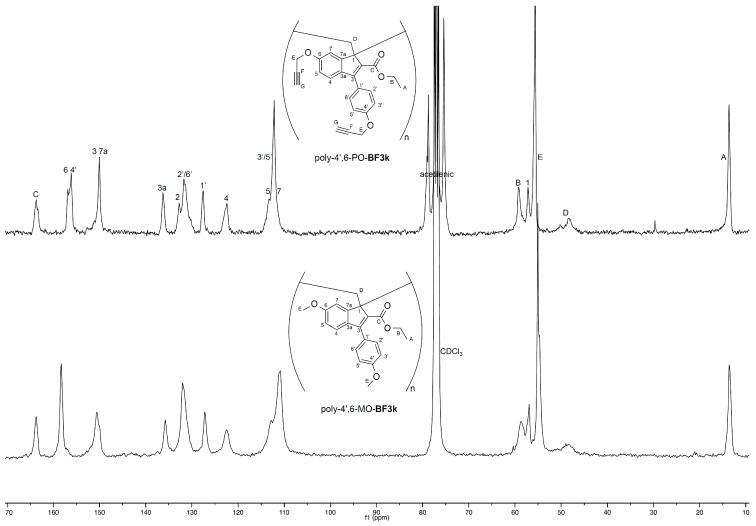
^13^C NMR spectrum (CDCl_3_) of poly-4’,6-PO-**BF3k** (**top trace**) compared with that of previously reported poly-4’6-MO-**BF3k** (**bottom trace**).

Previous ^13^C NMR studies carried out with poly-6-PO-**BF3k** suggested that the presence of propargyloxy groups in position 6 of the indene nucleus did not affect the vinyl (1,2) polymerization mechanism [28]. In order to evaluate the effects of the insertion of the second propargyloxy group on the structure of poly-4,6-PO-**BF3k** and poly-4’,6-PO-**BF3k**, the ^13^C NMR spectra of the these two polybenzofulvene derivatives were compared with that of poly-6-PO-**BF3k** (Figure 6). The intensive NMR studies performed on the members of the polybenzofulvene family allowed us to propose the chemical shifts of some signals in ^13^C NMR spectra as diagnostic, to evaluate the retention of the main vinyl-type polymerization. In particular, these signals correspond to C-1 (i.e., the aliphatic quaternary carbon adjacent to the methylene bridge D, at around 58 ppm); C-D (i.e., the carbon of methylene group acting as a bridge among the monomeric units along polybenzofulvene backbone, at around 48–51 ppm); C-1’ and C-3 (i.e., the carbons of the indene nucleus that may be affected by the competing 1,4-polymerization) [14]. The comparison shown in Figure 6 suggests the existence of an evident correspondence between poly-4’,6-PO-**BF3k** spectrum with that of poly-6-PO-**BF3k** in the up-field region containing backbone C-1 and C-D signals. This observation suggested the retention of the spontaneous 1,2-polymerization mechanism in these benzofulvene carboxylate derivatives. However, the ^13^C NMR spectrum of poly-4,6-PO-**BF3k** showed broad peaks, which appeared to be of difficult comparison with those shown by poly-4’,6-PO-**BF3k** and poly-6-PO-**BF3k**. Therefore, the spectra of poly-4,6-PO-**BF3k** were compared with those obtained with the previously published poly-4,6-MO-**BF3k** [13], bearing two methoxy groups in place of the two propargyloxy ones (Figure 7 and Figure 8). The comparison showed the stringent analogies expected from the assumed structural similarity, and suggested that the broadness of the signals in the ^13^C NMR spectra may depend on the positions occupied by the substituents rather than the nature of them. This assumption was confirmed by the correspondent comparison shown in Figure 9, where the ^13^C NMR spectrum of poly-4’,6-PO-**BF3k** is compared with the one of poly-4’,6-MO-**BF3k**.

#### 3.2.2. MALDI-TOF Mass Spectrometry

**Figure 10:**
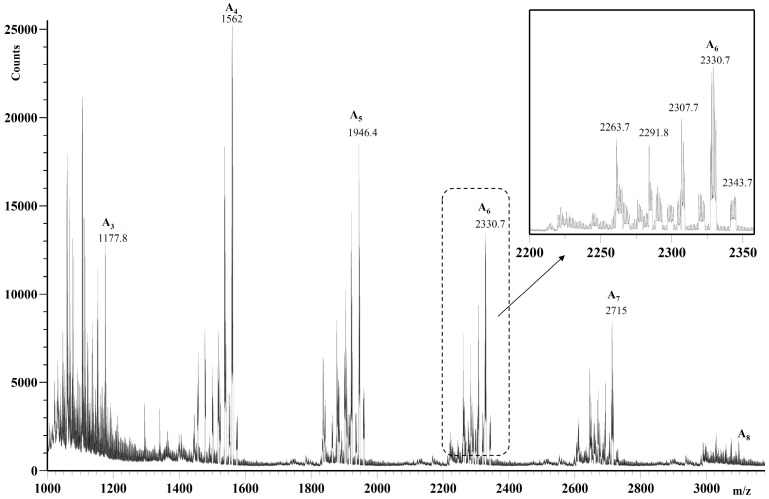
MALDI-TOF mass spectrum of poly-4,6-PO-**BF3k**.

The structure of poly-4,6-PO-**BF3k** and poly-4’,6-PO-**BF3k** was further characterized by MALDI-TOF mass spectrometry. Complex mass spectra were obtained in the case of poly-4,6-PO-**BF3k**, and a section of the one recorded using α-cyano-4-hydroxycinnamic acid (α-CHCA) as a matrix is displayed in Figure 10.

The mass spectrum presents a series of homologous peaks having 384.3 ± 0.2 Da transitions, corresponding to the mass of the repeating unit of poly-4,6-PO-**BF3k**. In particular, each cluster is constituted of the molecular ions M^+^ (i.e., 2307.7 ± n∙384.4) and sodiated ions (M + Na^+^) (i.e., peaks 1777.8, 1562, 1946.4, 2330.7, 2715 ± n∙384.4) and, also, of the ones due to the fragmentation process as reported previously [17]. Thus, the mass spectra confirmed the formation of the polybenzofulvene derivatives.

**Figure 11:**
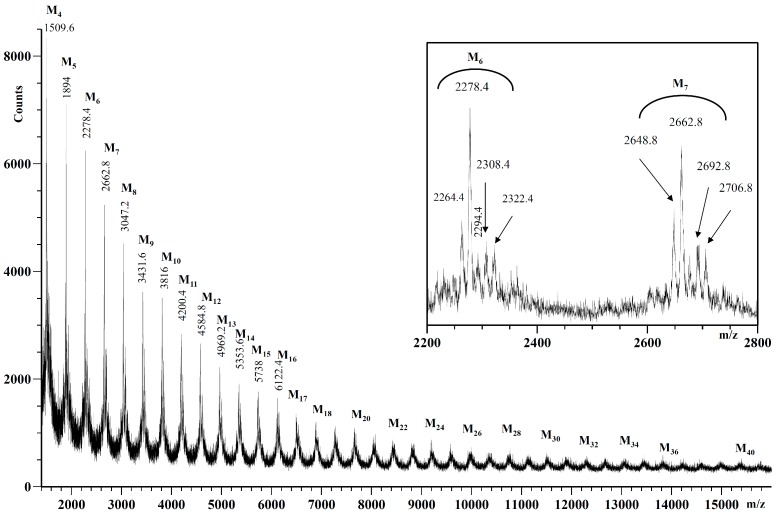
MALDI-TOF mass spectrum of poly-4’,6-PO-**BF3k**.

Figure 11 reports the MALDI-TOF mass spectrum of poly-4’,6-PO-**BF3k** recorded in linear mode using 2-(4-hydroxyphenylazo)benzoic acid (HABA) as a matrix and CF_3_COONa as a doping agent.

The spectrum shows, in the mass range *m*/*z* 1400–16,000 Da, a series of clusters of homologous peaks differing by 384.4 Da (the mass value of the monomeric unit) and corresponding to poly-4’,6-PO-**BF3k** macromolecular chains (4- to 40-mers). Thus, the mass spectrum confirms the formation of the polybenzofulvene derivative poly-4’,6-PO-**BF3k**. However, the analysis of the inset in Figure 11 suggests that each cluster was formed by the fragmentation mechanisms occurring during the MALDI-TOF analysis, as already observed in similar polybenzofulvenes [17].

#### 3.2.3. Wide-Angle X-ray Diffraction Studies

**Figure 12:**
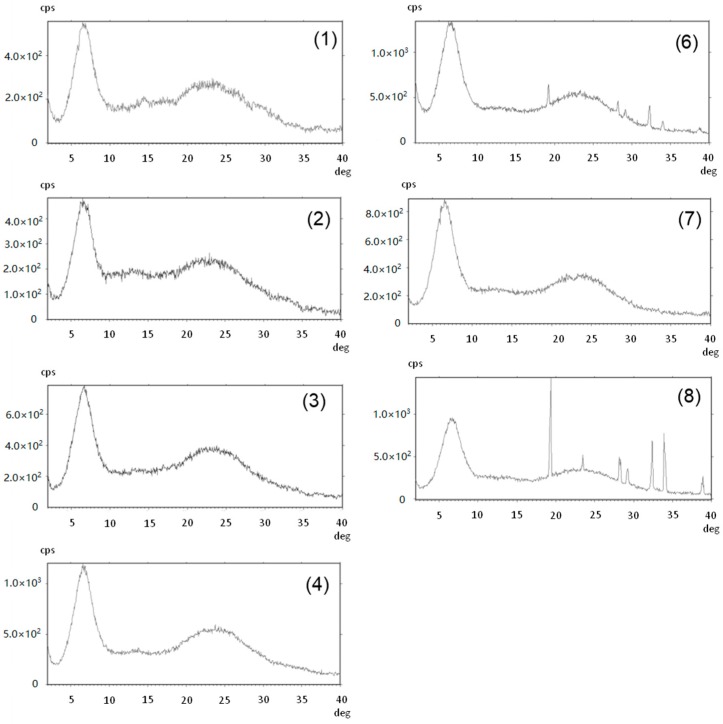
Wide-angle X-ray diffraction (WAXD) measurements performed in powder samples of poly-4’,6-PO-**BF3k** belonging to seven different batches of the polymer. The number between parentheses denotes the batch of the polymer as shown below in the Table 1.

Wide-angle X-ray diffraction (WAXD) measurements were performed in powder samples of poly-4’,6-PO-**BF3k** obtained by nanoprecipitation with methanol at room temperature from a polymer solution in chloroform. The results of WAXD analysis (Figure 12) performed on the samples belonging to seven different batches of the polymer, suggested very similar solid-state features, with the same broad peaks in all the analyzed samples, and the appearance of sharp signal patterns only in two samples.

#### 3.2.4. Molecular Weight Distribution Characterization

The characterization of the molecular weight distribution (MWD) in poly-4,6-PO-**BF3k** and poly-4’,6-PO-**BF3k** samples was performed by means of a multi-angle laser light scattering (MALS) detector on-line to a size exclusion chromatography (SEC) system with THF as the mobile phase.

The results obtained are summarized in Table 1 and suggested a quite reproducible MWD (*M*_p_ in the range of 275–396 kg/mol and polydispersity index *M*_W_/*M*_n_ in the range of 2.5–3.8) for poly-4,6-PO-**BF3k**, bearing the two propargyloxy groups on the same benzene ring. On the other hand, we realized that the spontaneous polymerization of 4’,6-PO-**BF3k** bearing the two propargyloxy groups in the two different benzene rings produced more variable results in terms of MWD of the corresponding polybenzofulvene derivatives. Thus, a number of poly-4’,6-PO-**BF3k** samples were prepared from different starting amounts of the monomers and their MWD was observed to span two orders of magnitude without an apparent reason. In particular, one sample reached the extraordinarily high value of 3444 kg/mol, whereas another sample showed a relatively low *M*_p_ value (33.3 kg/mol). While the relatively low molecular weight is unexplained, the aromatic T-shaped interaction among the pendant phenyl groups observed in the analysis of the crystal packing indenone derivative **9** could account for the ultrahigh molecular weight, when a similar interaction could be assumed to stabilize both the columnar aggregate, preluding the incipient polymer and the polymer itself. It is noteworthy that poly-4’,6-PO-**BF3k** samples showed, beside the very different polymerization degree, also different solubility in the mobile phase, as reflected by the different observed recovery values. Since the lowest recovery values were observed in two sample showing medium apparent *M*_p_ values, we assumed that different order degrees (i.e., crystallinity) could affect the solubility of poly-4’,6-PO-**BF3k** samples.

As expected, the radius of gyration (*R_g_*) was quite different depending on molecular weight and polymer conformation. For many samples, the conformation plot (*R_g_* = *K·M*^α^) was substantially linear with slope α value around 0.5, typical of a random coil polymer in a good solvent.

### 3.3. Structure of PPBFB Poly-4,6-MOEG-9-TM-**BF3k** and Poly-4’,6-MOEG-9-TM-**BF3k**

#### 3.3.1. NMR Spectroscopy

**Figure 13:**
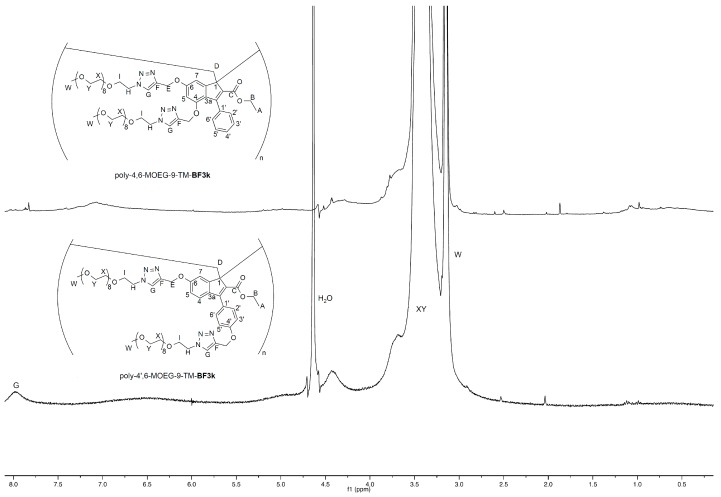
Comparison between the ^1^H NMR spectra of poly-4,6-MOEG-9-TM-**BF3k** (D_2_O, with water suppression) and poly-4’,6-MOEG-9-TM-**BF3k** (D_2_O).

The ^1^H NMR spectra of poly-4,6-MOEG-9-TM-**BF3k** and poly-4’,6-MOEG-9-TM-**BF3k** (Figure 13) were dominated by the signals at around 3.4 ppm, attributed to the MOEG-9 side chain in agreement with the presence of two side chains each monomeric unit. The spectra of these two PPBFB derivatives showed close similarities in the aliphatic regions. On the other hand, significant differences were observed in the aromatic regions due to the differences in the positions of the electron donating groups, as already observed for the precursor polymers poly-4,6-PO-**BF3k** and poly-4’,6-PO-**BF3k**.

**Figure 14:**
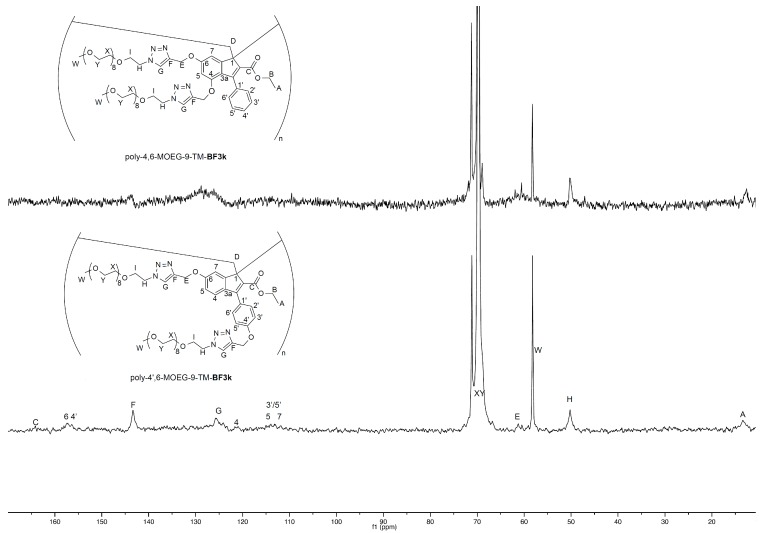
Comparison between the ^13^C NMR spectra of poly-4,6-MOEG-9-TM-**BF3k** (D_2_O) and poly-4’,6-MOEG-9-TM-**BF3k** (D_2_O).

The presence of the signal at around 8 ppm, attributed to the triazole hydrogen atom, supported the occurrence of the click chemistry grafting reaction, as further confirmed by the presence of the signals at 125.6 and 143.3 ppm in the ^13^C NMR spectrum (Figure 14) assigned to C–G and C–F of the triazole moieties.

#### 3.3.2. MALDI-TOF Mass Spectrometry

**Figure 15:**
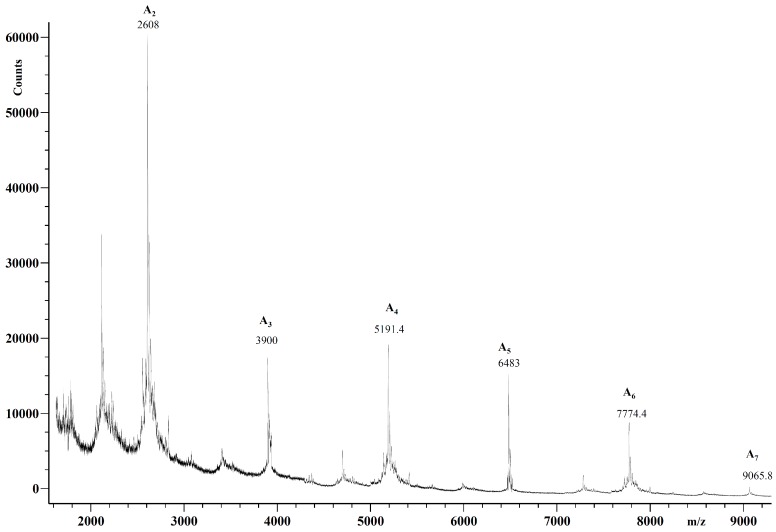
MALDI-TOF mass spectrum of poly-4,6-MOEG-9-TM-**BF3k**.

The structure of poly-4,6-MOEG-9-TM-**BF3k** and poly-4’,6-MOEG-9-TM-**BF3k** was also studied by MALDI-TOF mass spectrometry. The MALDI-TOF mass spectrum of poly-4,6-MOEG-9-TM-**BF3k** (Figure 15) was obtained in linear mode by using *trans*-2-[3-(4-*t*-butyl-phenyl)-2-methyl-2-propenylidene]malononitrile (DCTB) as the matrix and CF_3_COONa as a doping agent.

The mass spectrum showed a series of repeating families of peaks spanning from *m*/*z* 1500 up to *m*/*z* 9300 having 1291.4 ± 0.4 Da transitions, corresponding to the mass of the repeating unit of poly-4,6-MOEG-9-TM-**BF3k**. The most intense peaks corresponded to the sodiated ions of the expected oligomers terminated with hydrogens at both ends (species A_n_ in Figure 15).

**Figure 16:**
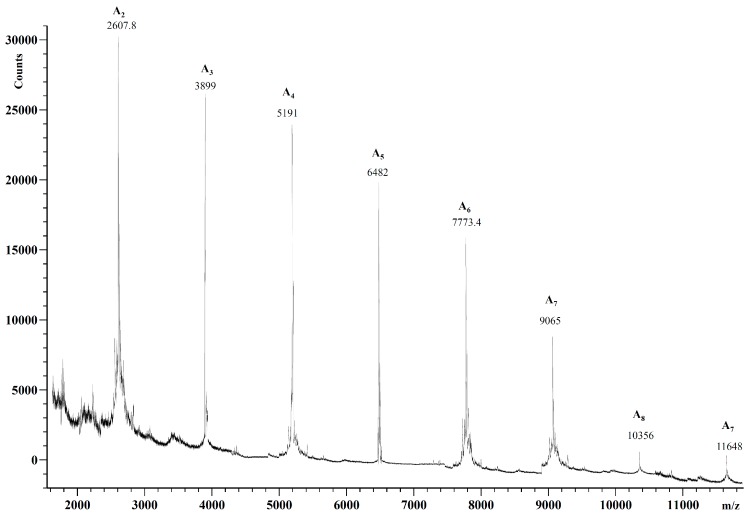
MALDI-TOF mass spectrum of poly-4’,6-MOEG-9-TM-**BF3k**.

Figure 16 shows the MALDI-TOF mass spectrum of poly-4’,6-MOEG-9-TM-**BF3k** obtained in linear mode by using malononitrile as a matrix, CF_3_COONa as doping agent, and DMF as the solvent.

The MALDI mass spectra of the poly-4’,6-MOEG-9-TM-**BF3k** confirmed the formation of the polymer, since it showed clusters of peaks equidistant 1291.4 ± 0.3 Da corresponding to the mass of the poly-4’,6-MOEG-9-TM-**BF3k** repeating unit. The most intense peaks were assigned to the sodiated ions of the oligomers terminated with hydrogen at both ends (species A_n_).

#### 3.3.3. Molecular Weight Distribution Characterization

The characterization of the molecular weight distribution in poly-4,6-MOEG-9-TM-**BF3k** sample was performed by means of a SEC-MALS system with THF/DMF (8:2) as the mobile phase. The results obtained (Table 2) confirmed the MWD features observed with the previously published PPBFB included in Table 2 for comparative purposes [25,26,28]. In fact, the MWD of poly-4,6-MOEG-9-TM-**BF3k** sample was characterized by *M*_p_ and polydispersity index *M*_W_/*M*_n_ values in the same range observed for poly-6-MOEG-9-TM-**BF3k** bearing only one MOEG-9 side chain in each monomeric unit.

### 3.4. Aggregation Features of Poly-4,6-MOEG-9-TM-**BF3k** and Poly-4’,6-MOEG-9-TM-**BF3k**

#### 3.4.1. Dynamic Light Scattering Analysis

PPBFB derivatives poly-4,6-MOEG-9-TM-**BF3k** and poly-4’,6-MOEG-9-TM-**BF3k** show peculiar architecture features that make them deemed among polymeric materials potentially useful in drug delivery applications. In fact, some polybenzofulvene molecular brushes have been demonstrated interesting features in complexing high molecular weight bioactive molecules such as immunoglobulin G (IgG) [29]. In these complexes, the protein–polymer interaction was assumed to occur by non-specific adsorption onto the surface of the hydrogel particles. Moreover, other PPBFB derivatives showed the capability of loading and delivering doxorubicin to cancer cells [30,34,35,36]. The carrier features of PPBFB depend on the structure and composition of the polymeric material, so that variations of these characteristics should modify its self-assembling properties, loading capacity and targeting properties.

The capability of PPBFB derivatives poly-4,6-MOEG-9-TM-**BF3k** and poly-4’,6-MOEG-9-TM-**BF3k** of generating supramolecular carriers spontaneously was investigated by means of dynamic light scattering analysis and zeta potential measurements. Thus, samples of poly-4,6-MOEG-9-TM-**BF3k** and poly-4’,6-MOEG-9-TM-**BF3k** were dispersed in ultrapure water at room temperature left under magnetic stirring for 2 days until complete dispersion of the polymers. DLS measurements were performed after 2 and 20 days and no mean variation in size was observed during this time frame (Table 3). Moreover, Z-average and Z-potential values do not change within the tested concentration range.

**Figure 17:**
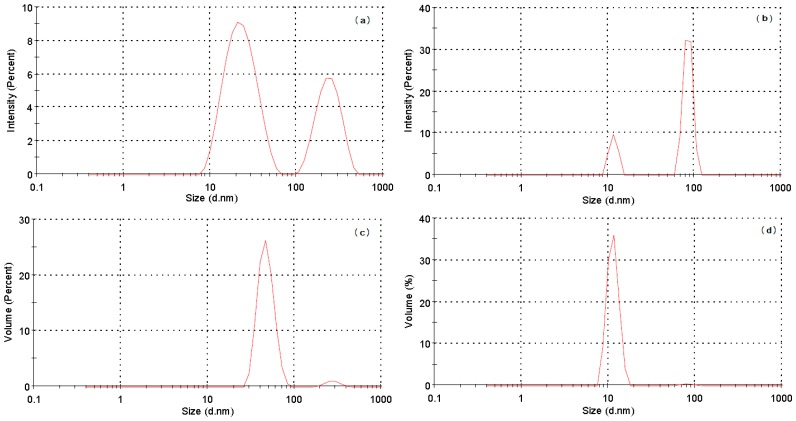
DLS size distribution histograms (by intensity **a** and **b**; by volume **c** and **d**) of poly-4’,6-MOEG-9-TM-**BF3k** (**a**,**c**) and poly-4,6-MOEG-9-TM-**BF3k** (**b**,**d**) dispersions in ultrapure water.

Interestingly, the results suggest that poly-4,6-MOEG-9-TM-**BF3k** is susceptible to producing water-soluble nanoparticles showing dimensions around 12 nm, in agreement with the gyration radius as determined by SEC-MALS experiments. Similarly, the results obtained with poly-4’,6-MOEG-9-TM-**BF3k** samples suggest that this PPBFB derivative was liable to generating water-soluble nanoparticles showing dimensions around 36 nm, which, on the bases of the apparent dimensions, were assumed to be isolated macromolecular nanoparticles. On the other hand, the relative tendency of these PPBFB derivatives to self-assemble in nanoaggregates was supported by the detection of supramacromolecular aggregates showing dimensions around 100 nm in the case of poly-4,6-MOEG-9-TM-**BF3k**, or around 240 nm in the case of poly-4’,6-MOEG-9-TM-**BF3k** (Figure 17).

#### 3.4.2. Transmission Electron Microscopy

**Figure 18:**
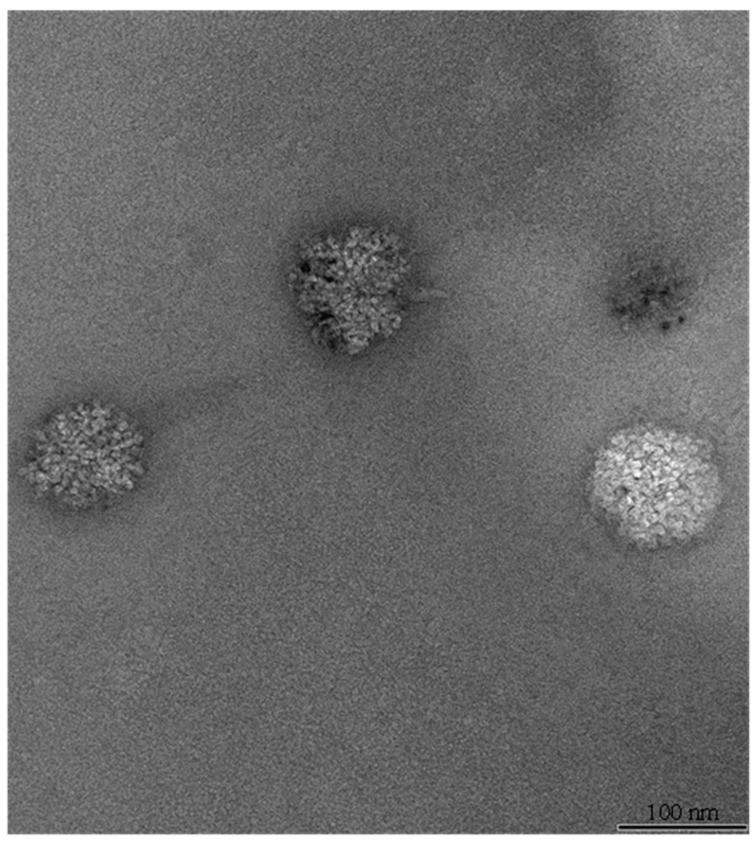
Structure of macromolecular aggregates found by TEM analysis of poly-4’,6-MOEG-9-TM-**BF3k** solutions in water. The scale bar corresponds to 100 nm.

The relative tendency of poly-4’,6-MOEG-9-TM-**BF3k** to self-assemble in nanoaggregates was confirmed by transmission electron microscopy (TEM) studies. In fact, solutions of this PPBFB derivative in water were observed by TEM after negative staining with uranyl acetate. TEM analysis revealed the presence of complex networks probably formed as the result of the probable collapse of isolated macromolecules (i.e., wormlike objects in agreement with the structure of cylindrical polymer brushes) or aggregates during the sample preparation for TEM experiments. However, the examination of a considerable number of microscopy fields allowed us to detect isolated macromolecular aggregates showing spherical shape and dimensions around 100 nm (Figure 18) resulting from the aggregation of a smaller population of particles. Thus, the information obtained by TEM images appeared to be in agreement with that provided by DLS experiments.

#### 3.4.3. Aggregation Mechanism

In general, PPBFB derivatives are amphiphilic macromolecules capable of interacting with a wide range of solvents. In particular, previously published poly-6-MOEG-9-TM-**BF3k** (obtained by means of the “grafting through” or “grafting onto” approaches) showed an excellent solubility in the most common organic solvents [22,23]. Its dissolution in dichloromethane or chloroform is very rapid, while the interaction with water is slower, but it led, however, to transparent solutions. These transparent solutions became cloudy when gently warmed above 35–37 °C, indicating that a significant hydrophobic collapse occurred and suggesting, for poly-6-MOEG-9-TM-**BF3k**, thermoresponsive features [14,27,28].

It is noteworthy that, in the same conditions, the dissolution of poly-4,6-MOEG-9-TM-**BF3k** was very rapid at room temperature, and the resulting solution was stable to pronounced heating. The comparison of this behavior with that observed with parent macromolecule poly-6-MOEG-9-TM-**BF3k** suggests, for the second water-solubilizing SC, a key role in aggregation properties.

**Figure 19:**
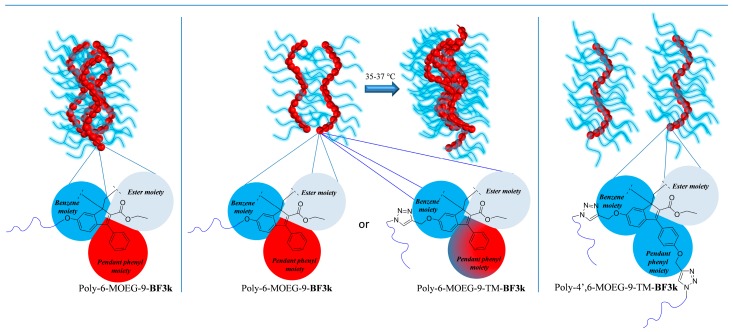
Possible aggregation mechanism in PPBFB derivatives.

Thus, the role of MOEG SCs in aggregation behavior of polybenzofulvene derivatives can be rationalized as follows. The first PEGylated member of the polybenzofulvene family poly-2-MOEG-9-**BF1** was found to generate quite stable aggregates, forming a compact physical hydrogel stable to the exposure to ultrasounds, because the attachment position of MOEG-9 SC leaves the most hydrophobic groups (the benzene moiety and the pendent phenyl ring) of the monomeric unit available for hydrophobic collapse and aggregation. In this PPBFB, the dissolution in water requires MOEG-9 SCs to be uniformly distributed in the 3D space along the polymer backbone with strict conformational constraints (Figure 19).

The shift of the attachment position to the benzene ring, as in poly-6-MOEG-9-**BF3k**, produces a decrease in aggregation liability (the hydrogel turns out to be sensitive to ultrasound exposure, which produces a disaggregation effect) because one of the crucial sectors of the monomeric unit for hydrophobic collapse becomes unavailable. The insertion of the TM spacer, as in poly-6-MOEG-9-TM-**BF3k**, is assumed to be capable of shifting the water-solubilizing moiety farther from the hydrophobic backbone, and of increasing the solvation shell around the PPBFB macromolecule. This produces an additional decrease in the aggregation liability, which reaches the maximum level in poly-4,6-MOEG-9-**BF3k** with the loss of the thermoresponsive behavior. In this polymer, as in poly-4’,6-MOEG-9-**BF3k**, the additional water-solubilizing moiety is assumed to be capable of shielding the pendent phenyl and of avoiding the thermo-induced hydrophobic collapse. From another point of view, the presence of the second MOEG-9 SC implies that suitable SC distribution around the backbone is reached with lower conformational constrains.

### 3.5. Biocompatibility of PPBFB Derivatives Poly-4,6-MOEG-9-TM-**BF3k** and Poly-4’,6-MOEG-9-TM-**BF3k**

**Figure 20:**
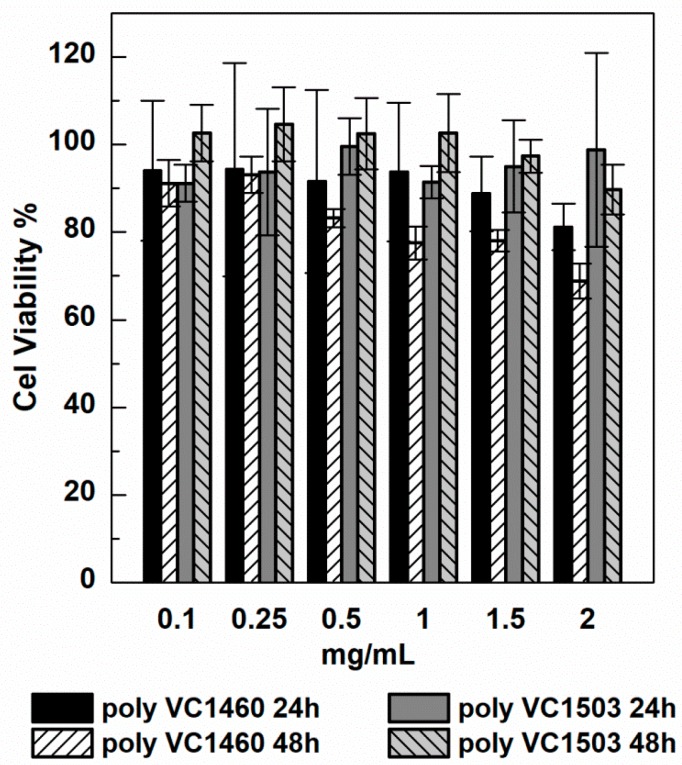
Cell viability % (MTS assay) of poly-4,6-MOEG-9-TM-**BF3k** (poly-VC1460) and poly-4’,6-MOEG-9-TM-**BF3k** (poly-VC1503) on human colon cancer HCT116 cells after 24 h and 48 h of incubation. Differences between tested groups are not statistically significant (*p* > 0.05).

The potential biocompatibility of PPBFB derivatives poly-4,6-MOEG-9-TM-**BF3k** and poly-4’,6-MOEG-9-TM-**BF3k** was assessed by evaluating their potential cytotoxicity on human colon adenocarcinoma HCT116 cells after incubation for 24 and 48 h. Several concentrations, ranging from 0.1 and 2.0 mg/mL, were assayed on the cell line at 24 and 48 h of incubation. The results shown in Figure 20 suggested that, for both incubation times, 24 h and 48 h, the cell viability was always comparable to the control. Therefore, these PPBFB derivatives were clearly cytocompatible in the whole concentration range tested, and may be potentially useful as nanocarriers for nanoencapsulation and delivery of drug molecules.

## 4. Conclusions

Two novel PEGylated polybenzofulvene brushes (i.e., poly-4,6-MOEG-9-TM-**BF3k** and poly-4’,6-MOEG-9-TM-**BF3k**) were prepared by means of a “grafting onto” approach, in which the polybenzofulvene backbone was assembled by spontaneous polymerization of the appropriate benzofulvene monomers bearing two clickable propargyloxy groups in different positions of the 3-phenylindene scaffold. Intermediate polybenzofulvene derivatives poly-4,6-PO-**BF3k** and poly-4’,6-PO-**BF3k** were thoroughly characterized by SEC-MALS, NMR spectroscopy, and MALDI-TOF MS techniques. Subsequently, their propargyloxy groups were exploited in the grafting with MOEG-9 chains by the most popular click chemistry (CuAAC) reaction, in order to obtain densely PEGylated polybenzofulvene brushes poly-4,6-MOEG-9-TM-**BF3k** and poly-4’,6-MOEG-9-TM-**BF3k**, which were characterized from the point of view of their macromolecular features, aggregation liability, and in a preliminary evaluation of biocompatibility.

Interestingly, DLS results obtained with poly-4’,6-MOEG-9-TM-**BF3k** samples suggested that this polymer was present in water solution mainly as isolated macromolecular nanoparticles (diameter around 20–40 nm) accompanied by rare supramacromolecular aggregates (diameter around 240 nm), highlighting a relative low tendency to self-assemble in nanoaggregates. This behavior was also confirmed by TEM studies. Therefore, in this polymer, the additional water-solubilizing moiety is assumed to be capable of shielding the pendent phenyl, and of avoiding the relative high hydrophobic interaction leading to aggregation observed in the previously reported PPBFB.

The modulation of the aggregation phenomenon, together with the intrinsic characteristic of double compartment (hydrophobic backbone and amphiphilic MOEG-9 side chains) and the cytocompatibility, make these PPBFB derivatives potentially useful as new materials for drug nanoencapsulation.

## Figures and Tables

**Table 1:** Macromolecular features of the newly-synthesized poly-4,6-PO-**BF3k** and poly-4’,6-PO-**BF3k** compared with those shown by previously reported poly-6-PO-**BF3k**, poly-6-MO-**BF3k**, and poly-**BF3k**.

Polymer (batch *n*)	*M*_p_^a^ (kg/mol)	*M*_W_^b^ (kg/mol)	*PDI* ^c^	*R*_g_^d^ (nm)	*K*^e^ (nm)	α ^e^	Recov. ^f^ (%)
poly-4,6-PO-**BF3k** (1)	396	693	3.8	45.0	0.0231	0.52	91
poly-4,6-PO-**BF3k** (2)	275	307	2.5	20.6	0.0196	0.53	98
poly-4’,6-PO-**BF3k** (1)	33.3	65.3	1.9	11.9	0.0336	0.48	91
poly-4’,6-PO-**BF3k** (2)	468	622	3.1	31.3	0.0223	0.51	87
poly-4’,6-PO-**BF3k** (3)	529	781	3.5	36.8	0.0218	0.51	83
poly-4’,6-PO-**BF3k** (4)	1059	961	5.7	45.0	0.0493	0.47	12
poly-4’,6-PO-**BF3k** (5)	1652	1348	3.3	48.5	0.0421	0.48	28
poly-4’,6-PO-**BF3k** (6)	1926	1351	3.0	46.1	0.0319	0.50	70
poly-4’,6-PO-**BF3k** (7)	2747	2375	2.7	42.8	0.0183	0.51	55
poly-4’,6-PO-**BF3k** (8)	3444	3118	2.4	79.9	0.0852	0.44	56
poly-6-PO-**BF3k**	2030	1794	2.2	51.5	0.0101	0.58	
poly-6-MO-**BF3k**	312	347	4.3	19.4	0.0058	0.61	
poly-**BF3k**	1900	1506	3.4	49.9	0.0066	0.60	

^a^*M*_p_: peak molecular weight; ^b^*M*_w_: weight-average molecular weight; ^c^*PDI*: polydispersity index *PDI* = *M*_w_/*M*_n_ where *M*_n_ denotes the number-average molecular weight; ^d^ *R*_g_: radius of gyration, which is the dimension of the macromolecules; ^e^ *K*, α: intercept and slope of conformation plot *R*_g_ = *KM***^α^**; ^f^ recov.: recovered mass, the fraction of the polymer eluted from the SEC column system.

**Table 2:** Macromolecular features of the newly synthesized poly-4,6-MOEG-9-TM-**BF3k** compared with those shown by previously reported poly-6-MOEG-9-TM-**BF3k**, poly-6-MOEG-9-**BF3k**, and poly-2-MOEG-9-**BF1**.

Polymer	*M*_p_^a^ (kg/mol)	*M*_W_^b^ (kg/mol)	*PDI* ^c^	*R*_g_^d^ (nm)	*K*^e^ (nm)	α ^e^	Recov. ^f^ (%)
poly-4,6-MOEG-9-TM-**BF3k**	121	279	6.4	22.3	0.0249	0.50	95
poly-6-MOEG-9-TM-**BF3k-GO**	484	821	8.9	33.6	0.0249	0.50	
poly-6-MOEG-9-TM-**BF3k-GT**	193	358	3.9	15.5			
poly-6-MOEG-9-**BF3k**	527	456	1.8	20.8			
poly-2-MOEG-9-**BF1**	74	175	3.0				

^a^*M*_p_: peak molecular weight; ^b^*M*_w_: weight-average molecular weight; ^c^*PDI*: polydispersity index *PDI* = *M*_w_/*M*_n_ where *M*_n_ denotes the number-average molecular weight; ^d^ *R*_g_: radius of gyration, which is the dimension of the macromolecules; ^e^ *K*, α: intercept and slope of conformation plot *R*_g_ = *KM*^α^; ^f^ recov.: recovered mass, the fraction of the polymer eluted from the SEC column system.

**Table 3:** DLS results obtained with poly-4,6-MOEG-9-TM-**BF3k** and poly-4’,6-MOEG-9-TM-**BF3k** in ultrapure water at 25 °C. Reported data are those recorded after 2 days (sample concentration 0.2 mg/mL).

Polymer Sample	Z-Average (nm)	PDI	Z-Potential (mV)
poly-4,6-MOEG-9-TM-**BF3k**	12	0.47	−14.8 ± 5.2
poly-4’,6-MOEG-9-TM-**BF3k**	36	0.34	−12.0 ± 5.4
